# A Monster in the Chest: A Tale of a Goiter

**DOI:** 10.7759/cureus.25827

**Published:** 2022-06-10

**Authors:** Sanjeev Sandasecra, Maya Mazuwin Yahya, Ahmad Zuhdi Mamat, Jien Yen Soh, Rosnelifaizur Ramely, Mohd E Aziz

**Affiliations:** 1 Department of Surgery, Universiti Sains Malaysia School of Medical Sciences, Kota Bharu, MYS; 2 Department of Radiology, Universiti Sains Malaysia School of Medical Sciences, Kota Bharu, MYS

**Keywords:** substernal goiter, sternotomy, thyroidectomy, mediastinum, superior vena cava syndrome

## Abstract

Substernal goiter is usually presented in elderly patients and is mostly asymptomatic. A large substernal goiter is surgically challenging and can be managed through a transcervical incision and sternotomy. This case report is about a large substernal goiter extending into the anterior mediastinum and causing superior vena cava syndrome that was resected via a transcervical and full sternotomy approach. The patient was a 47-year-old male, who visited our hospital for surgical treatment of substernal goiter. The computed tomography (CT) of the neck and thorax revealed a large substernal goiter extending into the mediastinum causing tracheal compression, vessel compression, and development of collateral vessels. Total thyroidectomy was performed via a full sternotomy and transcervical approaches. Postoperatively, the patient recovered well with no nerve palsy. Histopathological examination revealed the lesion as an adenomatous goiter. Substernal goiters are usually managed by transcervical approach, but a full sternotomy is required in cases of large substernal goiter with extension up to the pericardium and the presence of superior vena cava syndrome. A multidisciplinary team approach is necessary and can help reduce the risk of complications, such as nerve injury, major vessel injury, tracheal injury, and morbidity of the surgery.

## Introduction

A substernal goiter is commonly known as an extension of thyroid tissue into the mediastinum. The incidence of substernal goiter ranges from 5.1% to 15.7% in patients with thyroid disease and is commonly found in females than in males with a ratio of 4:1 [1-3]. Cervical goiter and substernal goiter are similar etiologically as both derive their blood supply from the neck region, especially the inferior thyroid artery. Descent of the cervical goiter through the thoracic inlet of more than 50% is a commonly accepted definition of substernal goiter [1].

The inferior extension of the goiter is due to anatomic constraints of the gland, which are demonstrated by limitation of the gland in all directions except the inferior. The gland is superiorly limited by the thyroid and cricoid cartilage, posteriorly limited by vertebral bodies with prevertebral fascia, and anteriorly limited by strap muscles and cervical fascia. The thyroid tissue from the cervical also grows downward because of the downward traction of swallowing, gravity, and negative intrathoracic pressure during respiration [2].

Goiters are slow-growing and may present for many years prior to presentation. Most patients are asymptomatic on presentation. In most cases, substernal goiters are operable via a cervical approach with cervical manipulation alone; however, in some cases, sternotomy is required, and a multidisciplinary team approach is considered. We report a case involving a large substernal goiter, which was safely resected via a full sternotomy and transcervical approach.

## Case presentation

A 47-year-old male visited our outpatient clinic with a complaint of anterior neck swelling for the past six years and increasing in size for the past two years prior to presentation. He complained of mild symptoms of discomfort at the anterior neck, with bulging veins and mild dyspnea. Otherwise, he denied any pain, dysphagia, hyperthyroid, or hypothyroid symptoms.

On examination, there was a large anterior neck swelling, and the left side was larger than the right side. The trachea was displaced to the right, extending inferiorly beyond the clavicle, and there was dullness on percussion up to the fourth intercostal cartilage bilaterally. There were enlarged superficial neck and upper chest veins suggestive of superior vena cava obstruction, but there was no numbness or weakness of the hands, no edema of the arms, no palpable lymph node, and no radial-to-radial delay of arterial pulsation.

**Figure 1:**
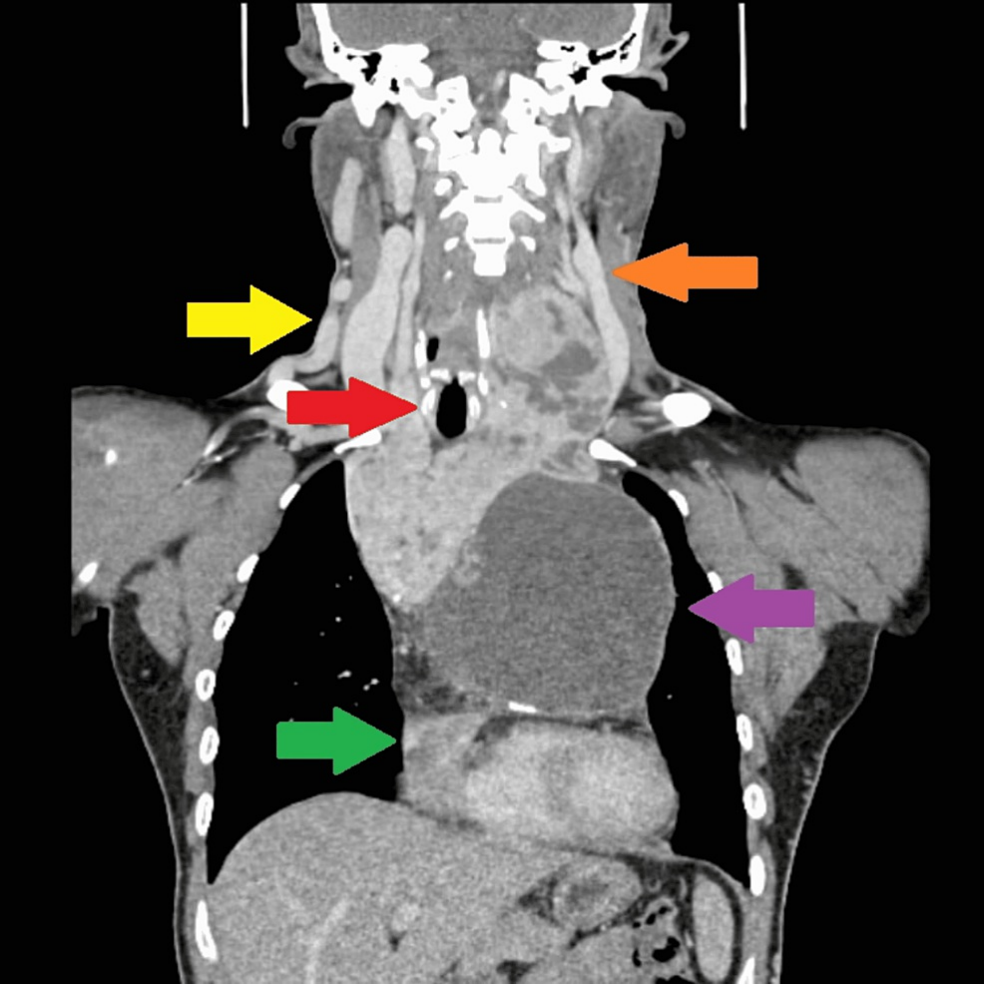
CT (coronal plane) of the thyroid gland with extension into the mediastinum. Yellow arrow indicates dilated right proximal external and internal jugular vein, orange arrow indicates dilated left internal carotid artery, red arrow indicates displaced trachea to right secondary to mass effect of goiter, purple arrow indicates cystic part of the goiter in the mediastinum compressing the heart, and green arrow indicates the compressed heart with clear plane between the pericardium and goiter.

**Figure 2:**
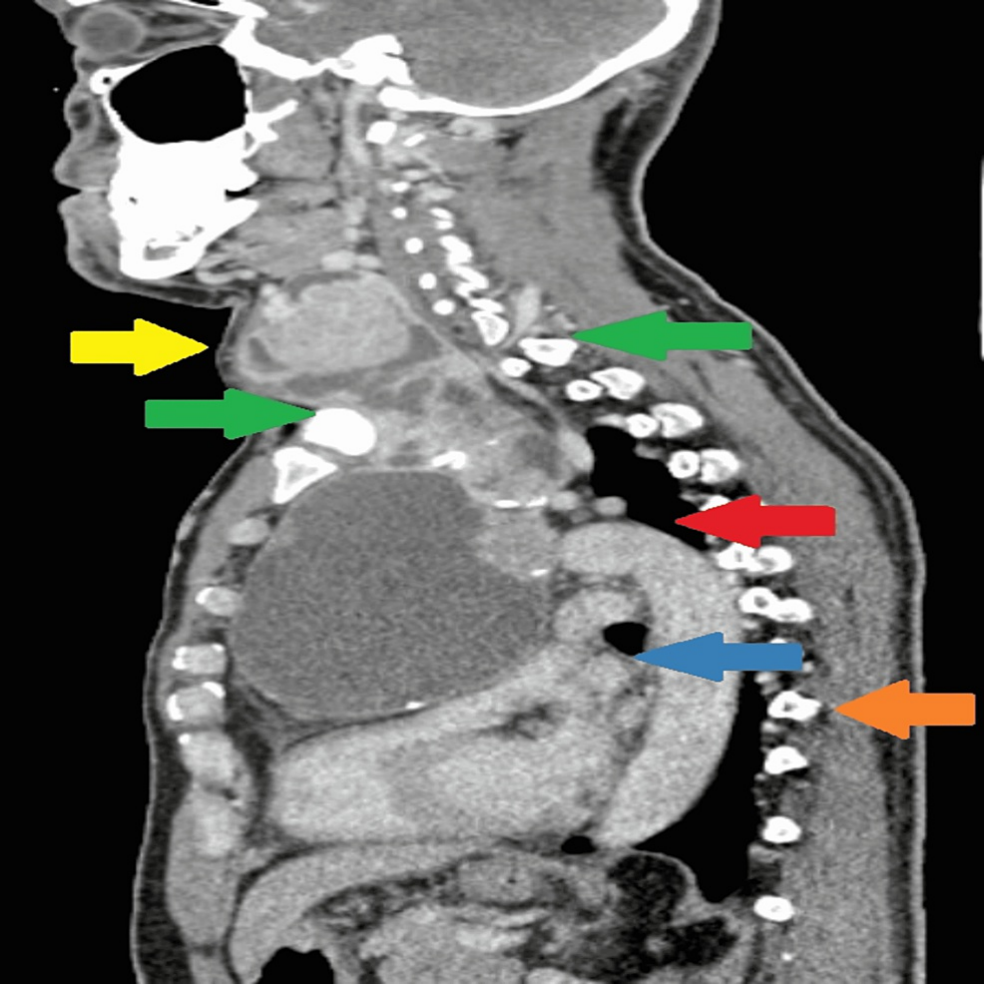
CT (sagittal plane) of the goiter extending below thoracic inlet into mediastinum. Yellow arrow indicates solid cystic component of goiter at the cervical region, green arrow indicates goiter extending below thoracic inlet, upper border of the manubrium (front) to upper border body of first thoracic (behind), red arrow indicates arch of the aorta, with the goiter extending below the arch of the aorta, blue arrow indicates the compressed superior vena cava, and orange arrow indicates goiter’s inferior border extending till T9 vertebra.

Routine laboratory tests revealed euthyroid status (thyroid-stimulating hormone: 1.14 mIU/L; free T4: 11.97 pmol/L). Thyroid ultrasound with computed tomography (CT) of the neck and thorax revealed that multiple mixed solid and cystic masses in both thyroid glands with extension into the mediastinum. The size of the right thyroid gland was 12.8 cm x 10 cm x 4.5 cm and the left gland measured 11.5 cm x 9.2 cm x 11.6 cm, with a large cystic lesion in the mediastinum measuring 11.5 cm x 9.2 cm x 12.1 cm and arising from the left thyroid gland. The right subclavian artery, brachiocephalic veins, and superior vena cava were compressed by the mass, and the intrathoracic part of the right jugular veins was severely compressed with dilated proximal jugular veins and collateral veins. The thyroid gland had large masses from both glands with extensive mediastinal extension and intrathoracic venous compression (Figures 1, 2).

Fine needle aspiration (FNA) cytology was performed in this case to exclude malignancy in view of the sudden increase in the thyroid size in the past two years. Biopsy showed a benign follicular nodule (Thy2), and indirect laryngoscopy showed left arytenoid prolapse, vocal cord medialization to the right side, and thinning of the left side vocal cord, but both were mobile. The result of laryngoscope was consistent with the FNA finding, and the thyroid swelling was benign.

The patient’s case was discussed in a multidisciplinary meeting involving an endocrine surgeon, cardiothoracic surgeon, vascular surgeon, otorhinolaryngology surgeon, and cardiac anesthesia team. The decision was made for a cervical and full sternotomy approach with tracheostomy because of the anticipated risk of tracheomalacia and respiratory compromise.

**Figure 3:**
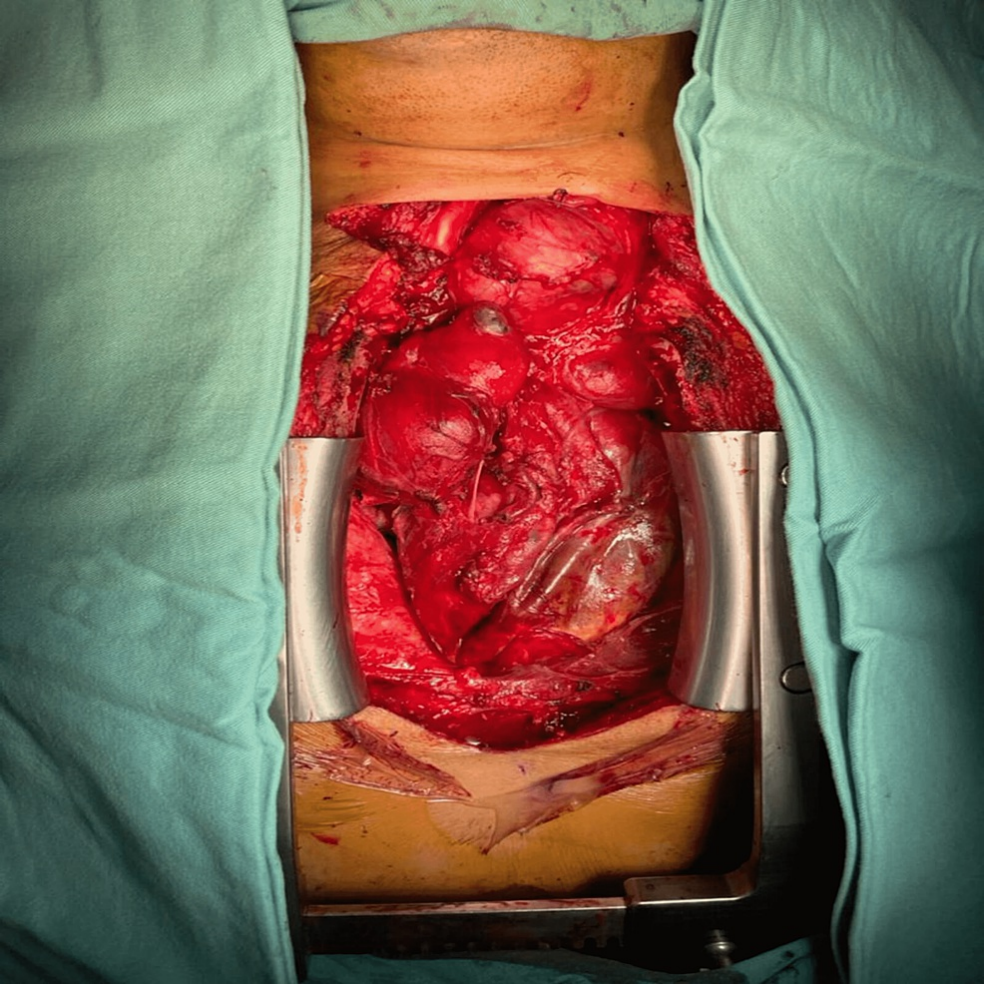
Intraoperative view of the goiter after sternotomy.

The patient underwent total thyroidectomy using a transcervical and full sternotomy approach. The anesthesiologist intubated the patient with a flexible bronchoscope. Sternotomy followed by the cervical skin incision was performed. The mediastinum component was tackled first. The goiter was well demarcated from the pericardium and lungs. The major blood vessels and nerves, including the phrenic nerves, the innominate vein, brachiocephalic trunks, superior vena cava, and right subclavian artery, were carefully separated from the substernal goiter, and then thyroidectomy was completed from the cervical incision (Figure 3).

**Figure 4:**
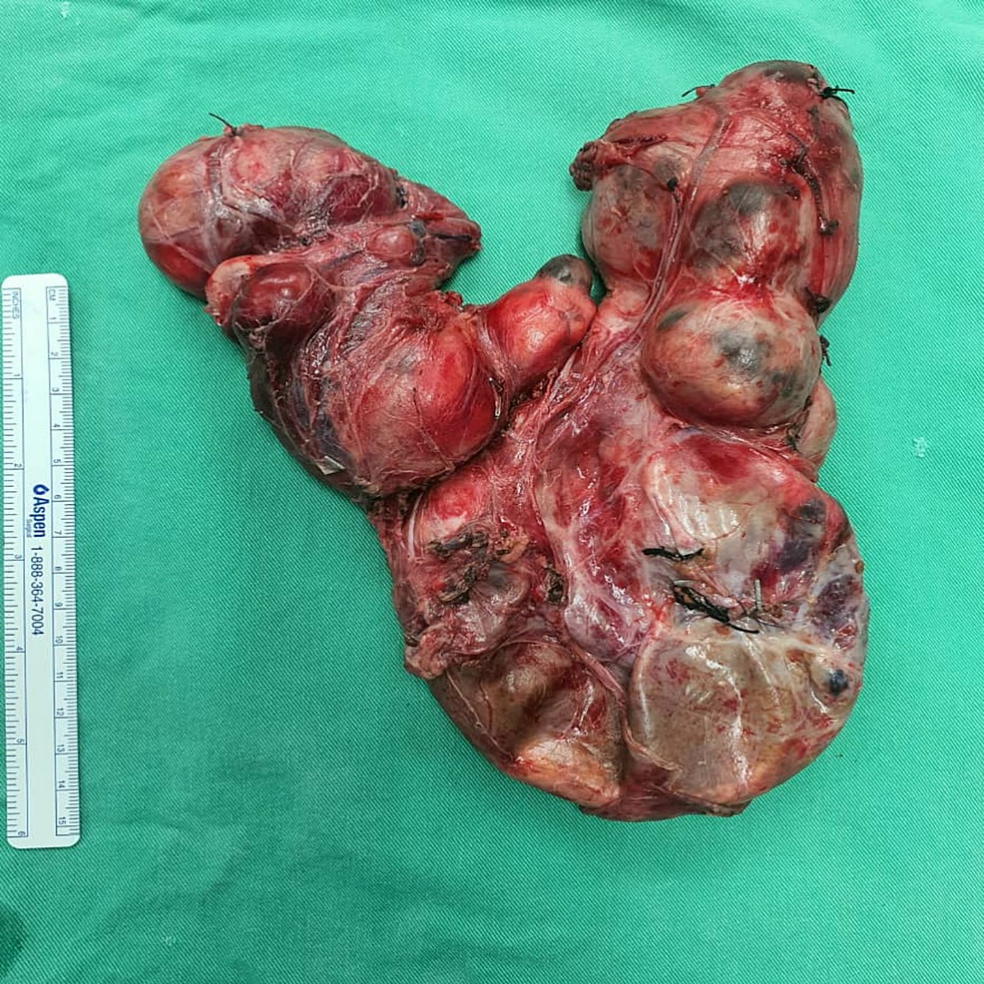
Resected goiter.

The left superior thyroid pedicle and left middle thyroid veins were ligated and dissected to allow the left thyroid to be rotated to gain a view of the recurrent laryngeal nerve (RLN) from the lateral aspect of the thyroid gland. The nerve monitoring probe was used to identify the nerve. The right thyroid lobe was more mobile than the left, and the same maneuver was performed on the right side. The parathyroid gland was identified and preserved. The substernal goiter that was connected to both the left and right thyroid glands was pulled in the cranial direction and entirely removed. The patient underwent tracheostomy to prevent any postoperative respiratory compromise. The wound was closed after drains were inserted into the neck and mediastinum. The patient was nursed in the surgical intensive care unit for observation. The total duration of the operation was 5 hours, and the total amount of intraoperative blood loss was 1,000 mL. The resected thyroid weighed 670 g (Figure 4).

**Figure 5:**
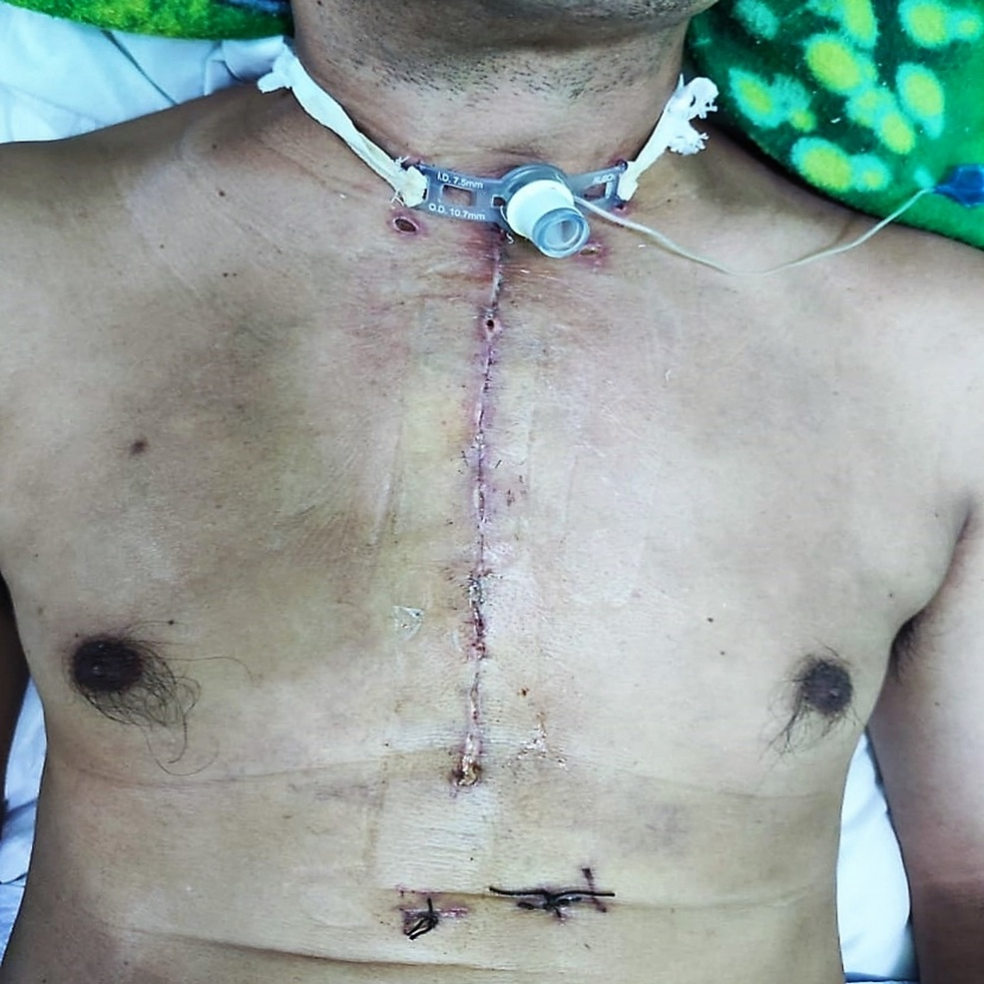
Postoperative wound. The patient was on tracheostomy.

Postoperative transient hypocalcemia was observed. Routine treatment with calcium supplement and L-thyroxine 0.1 mg was started. The patient was discharged home on day 5 following surgery with tracheostomy tube care and daily L-thyroxine. The histopathological examination showed adenomatous nodules within nodular hyperplasia. The tracheostomy tube was removed on day 30 post-surgery. The patient was well on his outpatient clinic visit, with no vocal cord palsy, and compliant with L-thyroxine (Figures 5).

## Discussion

The definition of substernal goiter varies and it has numerous names, such as retrosternal goiter and intrathoracic goiter. The frequency of substernal goiter vary due to difference in these definitions, and the most accepted current definition of substernal goiter is a goiter in which over 50% lesions are located in the thorax and the lesions extend below the thoracic inlet and 3 cm or more below the sternal notch or below the fourth thoracic vertebra [1-5]. In our patient, the goiter extended below the thoracic inlet, and the inferior border was at the level of the ninth thoracic vertebra.

The substernal goiter is more common in females compared to males, with a 1.6 times greater frequency, and the mean age of developing it is usually in the sixth decade of life [2,3,6]. Most substernal goiter, 80-90%, are in the anterior mediastinum, and 10-15% are in the posterior mediastinum [2]. Substernal goiters displaying unilateral expansion are more common than those with bilateral extension [3-5]. In our case, the patient was male and developed the goiter during his fourth decade of life; the large substernal goiter had a bilateral extension with more growth on the left, and this is uncommon because the extension to the right is more common because of the relatively loose areolar tissue found in this region.

Preoperative CT work-up of the neck and thorax is important in the management of substernal goiter especially in determining the extension and shape of the goiter. Several evaluation systems have been reported, and CT-based classification incorporating three spatial dimensions, anteroposterior (axial), laterolateral (coronal), and craniocaudal (sagittal) planes, have assisted in surgical planning for substernal goiter [7]. Information gained from CT scan is used for surgical planning, especially in our case. A multidisciplinary meeting was held to discuss the surgical approach that included the cardiothoracic team, the otolaryngology team, and a cardiac anesthesiologist to offer the patient an optimal recovery and safe surgery. Decision was made that patient does not need bypass since there were clear planes between the vessels and goiter. In this case, our patient had an enlarged superficial neck and upper chest veins suggestive of superior vena cava obstruction and evidence of collateral venous circulation on CT due to the goiter. The classification and approach for retrosternal goiter can be classified into grades 1, 2, and 3. These grades are given according to the anatomical location of the goiter, with grade 1 being above the aortic arch, grade 2 aortic arch to the pericardium, and grade 3 below the right atrium. These grades are also used for approach of surgery, such as cervical approach in grade 1, manubriotomy in grade 2, and full sternotomy in grade 3 [8]. Our patient goiter was graded as grade 3 as the goiter was compressing the heart, and hence the operative approach of full sternotomy was used.

The surgery was led by the endocrine surgeons, and the consensus is that substernal goiters are best managed surgically and that a cervical incision with full sternotomy will provide better exposure because it might not be possible to do so if the diameter of the goiter is over 10 cm or larger than the thoracic inlet [2,3]. The full sternotomy was preferred compared to ministernotomy as it gives sufficient exposure of the substernal goiter, and the surgery can be carried out in a simple and quick manner, with low morbidity rates [4].

Large substernal goiter can cause respiratory distress, vascular compression, dysphagia, and even sudden death. The substernal goiter carries a 3-21% risk of malignancy [4,5]. Surgery is the only safe and effective method in treating substernal goiter, even in healthy patients without clinical symptoms [4]. Surgical removal of a substernal goiter can often be done via a transcervical approach by skilled surgeons, especially endocrine surgeons; however, in certain cases, such as our patient, full sternotomy was indicated because of the large size of substernal goiter. It is reported that 29% of patients undergo sternotomy for substernal goiter [4,5]. In our case, surgery using the cervical and full sternotomy approach was adequate for total thyroidectomy, and additional incision such as thoracotomy was unnecessary because the substernal goiter was in the anterior mediastinum, and transcervical and the sternotomy approach allowed for better visualization and since the goiter had clear plane between vessels and pericardium and had abundant blood supply, such exposure was necessary for dissection and removal of the goiter from surrounding tissues. Postoperative complications such as major blood vessel injury and RLN injury were avoided.

The presence of substernal goiter is usually associated with slow growth of the cervical thyroid gland into the mediastinum. This process usually persists for over five years and is a long-standing condition. The duration can cause significant tracheal compression despite a patient being asymptomatic and is a risk factor for tracheomalacia and tracheostomy. Tracheomalacia with substernal goiter is an uncommon condition, but the risk of respiratory compromise still poses a threat to well-executed substernal goiter surgery [3,6]. In our case, tracheostomy was performed due to the long-standing tracheal compression and the abnormal findings of the preoperative vocal cord assessment. The tracheostomy tube was temporary and was removed 30 days post-surgery with full recovery and no RLN palsy. Temporary hypoparathyroidism occurs in patients after bilateral thyroid surgery, and ligation of thyroid vessel may cause temporary disruption to parathyroid blood supply leading to hypocalcemia postsurgery. Permanent hypocalcemia has been reported in 1-3% of patients [9]. Incidence of preserving the parathyroid gland is difficult in huge thyroid surgery especially due to its size; use of meticulous surgical technique, identification during surgery, and dissection close to the thyroid gland help in preventing removal of the parathyroid gland, and if parathyroid glands are damaged, autotransplantation of parathyroid gland should be performed.

## Conclusions

Substernal goiter remains one of the most underreported cases given that most patients present during the sixth or seventh decades of life and are asymptomatic. The approach for substernal goiter is individualized for each patient. The transcervical and full sternotomy incision gives a good exposure and is indicated when the goiter extends on both sides of the thorax and has a larger diameter than the thoracic inlet. The involvement of multiple teams in managing substernal goiter is essential in the current era of medicine, and the decision for surgery should be both a consensus to reduce the morbidity of surgery and in line with the World Health Organization’s guidelines for safe surgery safe lives.
